# Emotional Intensity Modulates the Integration of Bimodal Angry Expressions: ERP Evidence

**DOI:** 10.3389/fnins.2017.00349

**Published:** 2017-06-21

**Authors:** Zhihui Pan, Xi Liu, Yangmei Luo, Xuhai Chen

**Affiliations:** ^1^Key Laboratory of Behavior and Cognitive Psychology in Shaanxi Province, School of Psychology, Shaanxi Normal UniversityXi'an, China; ^2^State Key Laboratory of Cognitive Neuroscience and Learning and IDG/McGovern Institute for Brain Research, School of Brain Cognitive Science, Beijing Normal UniversityBeijing, China

**Keywords:** facial, vocal, anger, emotional intensity, integration, ERP

## Abstract

Integration of information from face and voice plays a central role in social interactions. The present study investigated the modulation of emotional intensity on the integration of facial-vocal emotional cues by recording EEG for participants while they were performing emotion identification task on facial, vocal, and bimodal angry expressions varying in emotional intensity. Behavioral results showed the rates of anger and reaction speed increased as emotional intensity across modalities. Critically, the P2 amplitudes were larger for bimodal expressions than for the sum of facial and vocal expressions for low emotional intensity stimuli, but not for middle and high emotional intensity stimuli. These findings suggested that emotional intensity modulates the integration of facial-vocal angry expressions, following the principle of Inverse Effectiveness (IE) in multimodal sensory integration.

## Introduction

Successful social interaction requires a precise understanding of the feelings, intentions, thoughts, and desires of other people (Sabbagh et al., 2004). Human beings have to integrate sensory cues came from facial expressions and vocal intonation to create a coherent, unified perception (Klasen et al., 2012), given that the evaluation of emotion is rarely based on the expression of one modality alone. Although, the phenomenon of bimodal emotion integration has been clearly depicted (Klasen et al., 2012; Chen et al., 2016a,b), the influence of emotional features thereof, such as emotional intensity, has seldom been tested. This is of importance as emotional intensity has been proved to be a key factor influencing emotional perception (Sprengelmeyer and Jentzsch, 2006; Yuan et al., 2007; Dunning et al., 2010; Wang et al., 2015) and the modulation of emotional intensity is one important way to test the principle of Inverse Effectiveness (IE), that is, multisensory integration is more effective when its constituent modality is less salient (Collignon et al., 2008; Stein and Stanford, 2008; Stein et al., 2009; Jessen et al., 2012). Therefore, the current study aims to test modulation of emotional intensity on bimodal anger integration using the technic of electroencephalography (EEG) with high temporal resolution.

The interaction of bimodal emotional cues, particularly the emotional signals delivered by facial and vocal expressions, has been widely reported (Klasen et al., 2012). One line of studies showed that bimodal emotions led to shorter response times and higher response accuracy, demonstrating a facilitation effect of bimodal emotional cues (Dolan et al., 2001; Klasen et al., 2012; Schelenz et al., 2013). Additionally, it was observed that bimodal emotional cues reduced the amplitude of the early auditory N1 compared to unimodal cues (Jessen and Kotz, 2011; Kokinous et al., 2015), while the redundant emotional information in the auditory domain reduced the amplitude of the P2 and N3 components compared to faces alone (Paulmann et al., 2009). These attenuated components were considered the neural substrate of facilitated perceptual processing (Klasen et al., 2012). Some other studies compared the brain responses for congruent bimodal emotions with those for incongruent bimodal emotions, and found that congruent bimodal emotions enhanced the N1 (De Gelder et al., 2002) and P2 (Balconi and Carrera, 2011) amplitudes relative to incongruent ones, suggesting an integration of bimodal emotional cues indirectly. Conversely, some other studies tested the bimodal integration directly, by comparing the bimodal responses with the summed unimodal responses, which results in either sub- or supra-additive response (Stein and Stanford, 2008; Hagan et al., 2009). For instance, Hagan et al. (2009) observed a significant super-additive gamma oscillation within the first 250 ms for congruent audiovisual emotional perception. Likewise, Jessen and Kotz (2011) found stronger beta suppression for multimodal than for the summed unimodal conditions. Moreover, recent evidence shows a robust superadditivity in P3 amplitudes (Chen et al., 2016a,b) and theta band power (Chen et al., 2016b) during bimodal emotional change perception.

Taken together, the interaction and/or integration of facial-vocal emotional cues have been well-examined. However, some studies argued that the principle of Inverse Effectiveness is the most valid way to test the multimodal integration (Stein and Stanford, 2008; Stein et al., 2009). Concerning the integration of multimodal emotions, it was reported that bimodal emotional cues affected each other, especially when the target modality was less reliable (Collignon et al., 2008) and multimodal emotions elicited earlier N1 than unimodal emotions under high noisy background but not low noisy background (Jessen et al., 2012). Nevertheless, all these studies tested IE principle by manipulating the noisy level of the background. To precisely delineate the integration of bimodal emotion integration, it is necessary to manipulating the saliency of the emotional stimulus itself. One of the effective ways to manipulating emotional saliency is changing emotional intensity, which has been proved to be a key factor influencing emotional perception (Sprengelmeyer and Jentzsch, 2006; Yuan et al., 2007; Dunning et al., 2010; Wang et al., 2015). For instance, Dunning et al. (2010) reported that as the percentages of anger in faces increased from 0 to 100%, faces were perceived as increasingly angry. Moreover, it was reported that the N170 amplitudes increased as the augment of the emotional intensity (Sprengelmeyer and Jentzsch, 2006; Wang et al., 2013). Likewise, it was found that the rates of happiness and P300 amplitudes increased as emotional intensity increased in vocal emotion decoding (Wang et al., 2015).

Therefore, the present study examined the integration of facial and vocal emotion cues by manipulating emotion intensity. Specifically, we asked participants to perform emotion identification task on facial, vocal, and bimodal emotion expressions varied in emotional intensity. As some studies (Yuan et al., 2007, 2008) indicated that emotional intensity effect is more prominent in negative emotions, only angry expressions were studied in the current study.

Based on the findings that emotional intensity modulates emotion processing in single modality (Sprengelmeyer and Jentzsch, 2006; Yuan et al., 2007; Dunning et al., 2010; Wang et al., 2015), and bimodal integration effect was more salient under high noisy background than under low noisy background (Jessen et al., 2012), we hypothesize that bimodal emotion integration effect was more conspicuous for stimuli with low emotional intensity stimuli than for those with high emotional intensity, following the principle of IE. As the integration effect was mainly denoted by N1 and P2 components (Paulmann et al., 2009; Balconi and Carrera, 2011; Jessen and Kotz, 2011; Jessen et al., 2012; Ho et al., 2015; Kokinous et al., 2015, 2017), we predict that the modulation of emotional intensity should be manifested on these components. To circumvent the confound of the physical difference in the direct comparison between bimodal and unimodal stimuli, we compare the brain responses associated bimodal emotions with the sum of those linked with both vocal and facial emotions (Brefczynski-Lewis et al., 2009; Hagan et al., 2009; Stevenson et al., 2012; Chen et al., 2016a,b). Based on the amplitude reduction of the early N1 component for bimodal emotions vs. unimodal emotions reported in previous studies (Jessen et al., 2012; Kokinous et al., 2015), we predicted that bimodal emotions should elicited reduced N1 relative to unimodal emotions. However, given the controversy regarding the P2 amplitude associated multimodal integration, specifically, while many studies reported a suppression P2 components in the bimodal compared with the unimodal condition (Pourtois et al., 2002; Paulmann et al., 2009; Kokinous et al., 2015), some other studies found an enhanced P2 components for bimodal relative to unimodal condition (Jessen and Kotz, 2011; Schweinberger et al., 2011), no prior hypotheses were made with regard to the increase or decrease of the P2 amplitude.

## Method

### Participants

Twenty-five university students (12 women, aged 19–27, mean 23.6 years) were recruited to participate in the experiment. All the participants reported normal auditory and normal or corrected-to-normal visual acuity, and were free of neurological or psychiatric problems. The study was approved by the Local Review Board for Human Participant Research and written informed consent was obtained prior to the study. The experimental procedure was in accordance with the ethical principles of the 1964 Declaration of Helsinki (World Medical Association Declaration of Helsinki, 1997). All participants were reimbursed ¥50 for their time. Five participants were excluded from EEG data analysis because of extensive artifacts.

### Materials

Angry and neutral expressions for “嘿/hei/” and “喂/wei/” were chosen from our emotional expression database (the development of the database can be found in the Supplementary Materials). Based on the original expressions, vocally neutral-angry continua (33, 67, and 100% anger) were created for each interjection using STRAIGHT (Kawahara et al., 2008), while facially neutral-angry continua were created using Morpheus Photo Morpher 3.16 (Morpheus Software LLC, Santa Barbara, CA, USA). And the bimodal expressions were created through presenting the vocal and facial expressions together. To verify the validity of the emotional intensity manipulation, 23 participants were asked to decide whether the synthesized expression expresses anger and to rate the emotional intensity level of the stimulus on a 9-point scale, with 1 being “not intense” and 9 being “highly intense.” The mean ratios of anger and intensity level scores are presented in Table 1. The repeated measures ANOVA with Intensity (33, 67, and 100%) and Modality (Facial, Vocal, and Bimodal) as within subject factors on ratios of anger and intensity level confirmed the validity of our emotional intensity manipulation. Specifically, the analysis on ratios of anger showed significant main effect of Intensity [*F*_(2, 44)_ = 53.32, *p* < 0.001, η^2^_*p*_ = 0.71], with the ratios of anger higher for 100% than for 67 and 33% (*p* < 0.01 and *p* < 0.001, respectively), and higher 67% than for 33% (*p* < 0.001). Similarly, the analysis on intensity levels showed significant main effect of Intensity [*F*_(2, 44)_ = 85.37, *p* < 0.001, η^2^_*p*_ = 0.80], and significant interaction between Modality and Intensity [*F*_(4, 88)_ = 9.76, *p* < 0.01, η^2^_*p*_ = 0.31]. Simple effect analyses showed that intensity level were larger for 100% than for 67 and 33% (*ps* < 0.001) under all modalities, and larger for 67% than for 33% under facial and bimodal conditions (*ps* < 0.01), while no difference was found between 67 and 33% under vocal condition (*p* > 0.1).

**Table 1:** Rates of response anger and Intensity level as function of emotional intensity (M ± *SD*).

	**Rates of response anger**			**Intensity level**		
	**Vocal**	**Facial**	**Bimodal**	**Vocal**	**Facial**	**Bimodal**
33%	0.41 ± 0.21	0.52 ± 0.27	0.61 ± 0.31	4.93 ± 1.25	4.28 ± 1.18	4.50 ± 1.12
67%	0.73 ± 0.17	0.73 ± 0.21	0.89 ± 0.15	5.05 ± 1.19	5.79 ± 1.21	5.71 ± 1.30
100%	0.89 ± 0.17	0.80 ± 0.13	0.93 ± 0.14	6.07 ± 1.31	6.46 ± 1.30	6.75 ± 1.22

## Procedure and design

**Figure 1:**
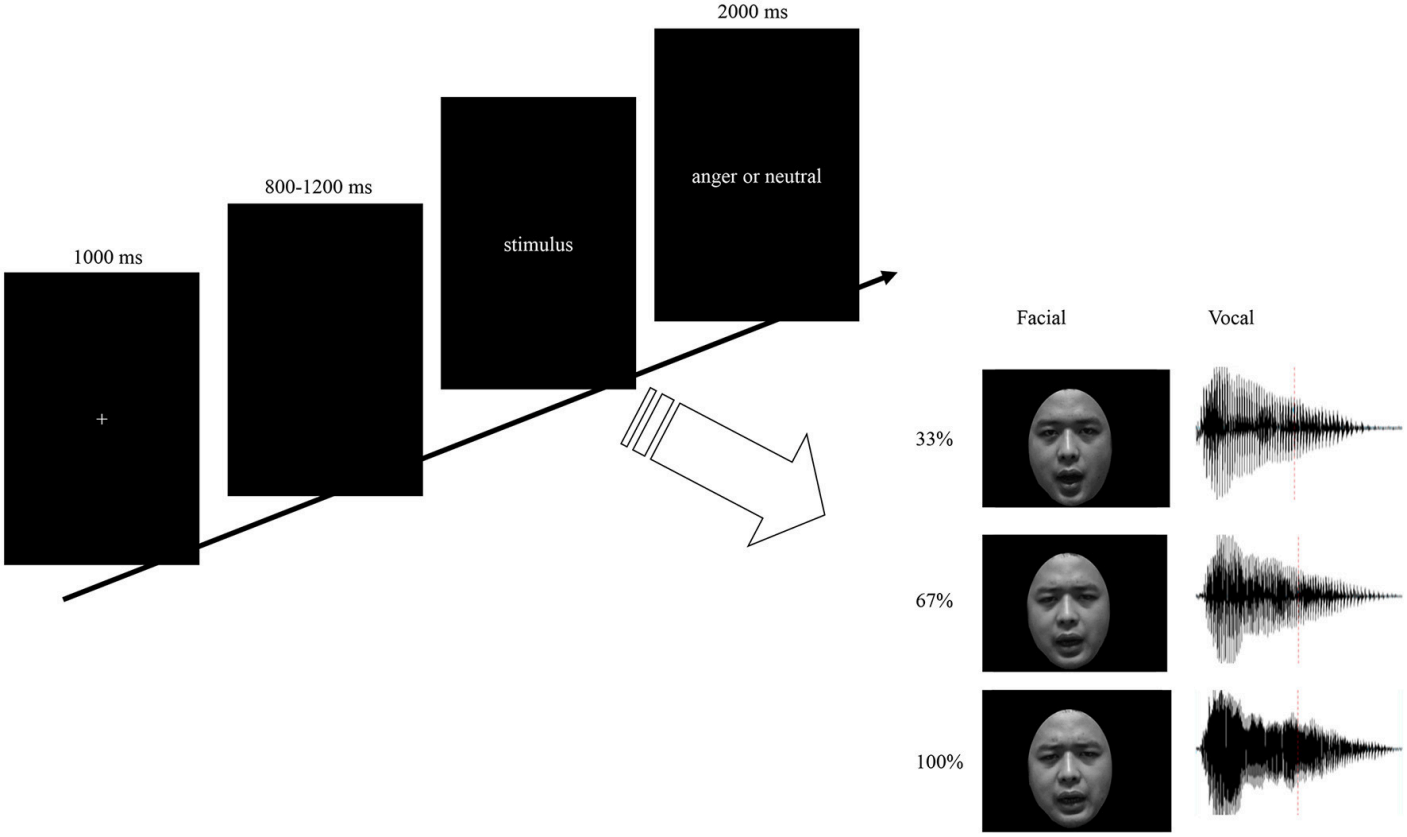
Schematic representation of stimuli and experimental design. Trials consisted of fixation, blank jitter, stimuli presentation, and decision. The facial stimuli were presented with same duration as the vocal stimuli. By orthogonally combine the modality (F, V, and FV) and intensity (33, 67, and 100%) factors, nine levels of stimuli were constructed.

Each participant was seated comfortably in a sound-attenuated room. Stimulus presentation was controlled using E-prime software. Auditory stimuli were presented via loudspeakers placed at both sides of the monitor while the facial stimuli presented on the monitor simultaneously at a central location on a black background (visual angle of 3.4° [width] × 5.7° [height] from a viewing distance of 100 cm). As illustrated in Figure 1, each trial was initiated by a 1,000 ms presentation of a white cross on the black computer screen. Then, a blank jitter varied randomly between 800 and 1,200 ms was presented, followed by the stimuli. Participants were required to decide whether the stimuli expressed anger by pressing the “J” or “F” button on the keyboard as accurately and quickly as possible. The response buttons assigned for “yes” or “no” was counterbalanced across participants. The presentation time for facial expressions and bimodal expressions were the same as its corresponding vocal expressions. Each response was followed by 2,000 ms of a blank screen. The stimuli consisted with facial, vocal and bimodal expressions varied in emotional intensity. Based on the orthogonal combination of 3 modals, 3 intensity levels, and 2 interjections, 18 emotional interjections were include. To enhance the signal to noise ratio, each stimulus was repeated 20 times, and therefore 360 emotional expressions were presented to participants as key stimuli. Moreover, to increase the ecological validity and balance the response of “yes” and “no” in the current study, 120 neutral expressions were presented to the participants as filler. The total 480 stimuli were randomized and split up into five blocks and participants were given a short self-paced break to rest between blocks. Pre-training with 48 trials was included in order to make subjects familiar with the procedure.

## EEG recording

Brain activity was recorded at 64 scalp sites using tin electrodes mounted in an elastic cap (Brain Product, Munich, Germany) according to the modified expanded 10–20 system, each referenced on-line to FCZ. Vertical electrooculograms (EOGs) were recorded supra-orbitally and infra-orbitally from the right eye. The horizontal EOG was recorded as the left vs. right orbital rim. The EEG and EOG were amplified using a 0.05–100 Hz bandpass and continuously digitized at 1,000 Hz for offline analysis. The impedance of all electrodes was kept <5 kΩ.

### Data analysis

#### Behavioral data

**Figure 2:**
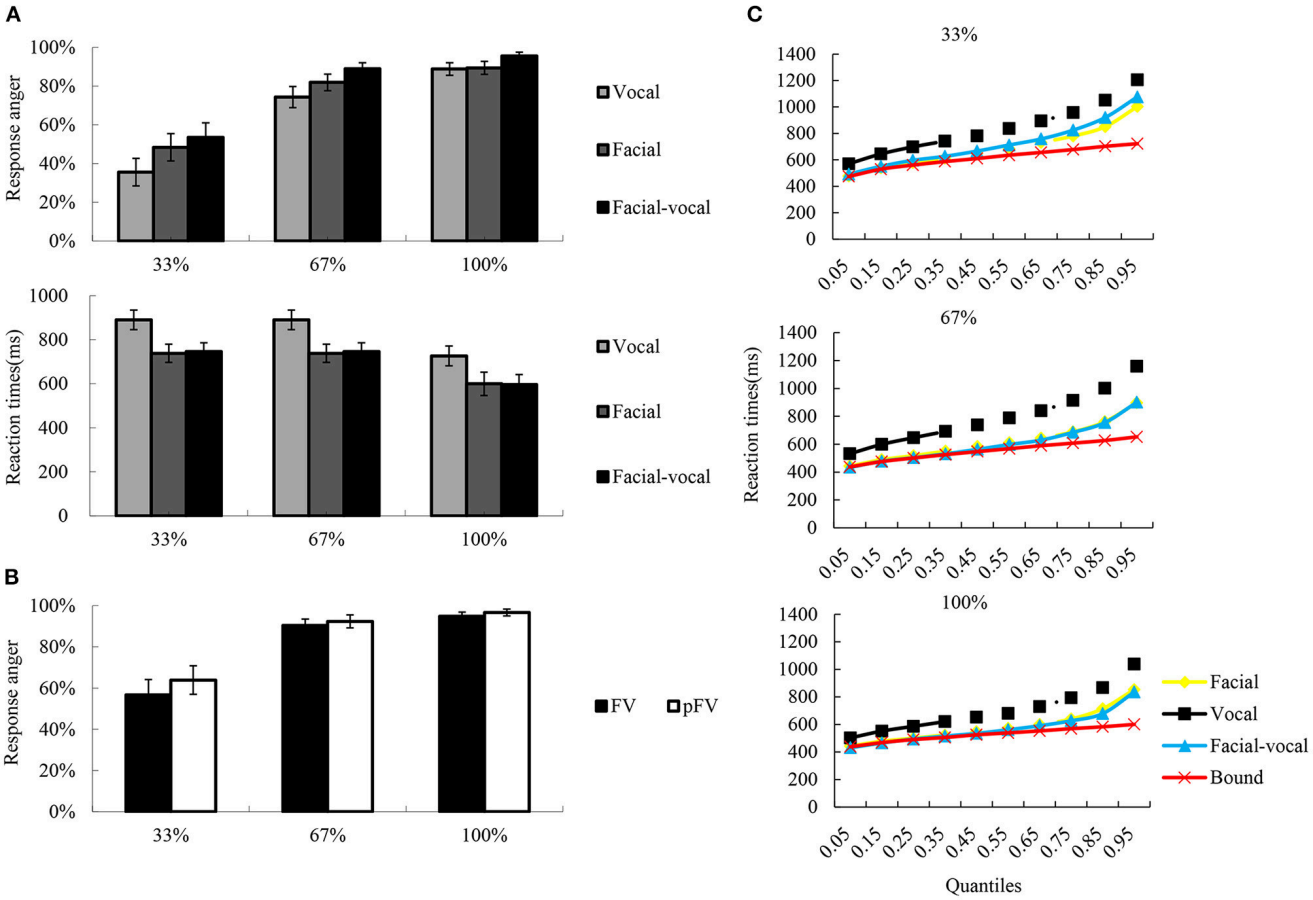
A schematic illustration of behavioral performance. **(A)** The bar char reflects the reaction times and the rates of response anger. Error bars indicate standard error. **(B)** The rates of response anger for FV and pFV at each intensity level, as well as the pFV based on unimodal condition. **(C)** Redundancy gain analysis and test for violation of the race model inequality. The scatter plots illustrate the cumulative probability distributions of the RTs (all quantiles are displayed) for the bimodal (blue triangles) and their unimodal counterparts (orange squares for facial, black rhombuses for vocal), as well as the race model bound (red crosses) computed from the unimodal distributions. The race model inequality was not significantly violated across all quantiles of the reaction time distribution since only bimodal values inferior to the bound indicated violation of the race model.

The rates of response anger and reaction times were collected as dependent variable, and were subjected to repeated measures ANOVA with *Modality* (F, V, and FV) and *Intensity* (33, 67, and 100%) as within-subject factors, to test the emotional intensity effect across modalities. To investigate the modulation of emotional intensity on bimodal integration, then, the predicted rates of response anger (pFV) were calculated following the equation [pFV = p(F) + p(V) − p(F)^*^p(V)] by Stevenson et al. (2014)^1^, and subjected to ANOVA with *Intensity* (33, 67, and 100%) and *Modality-type* (FV, pFV) as within subjects factors. And whether the RTs in the bimodal condition exceeded the statistical facilitation predicted by probability summation of two unimodal conditions (Miller, 1982) was tested using the algorithm implemented in RMITest software (Ulrich et al., 2007; see Figure 2 for graphic illustration). The degrees of freedom of the F-ratio were corrected according to the Greenhouse–Geisser method and multiple comparisons were Bonferroni adjusted. The effect sizes were shown as partial eta squared (η^2^_*p*_).

#### ERP data

**Figure 3:**
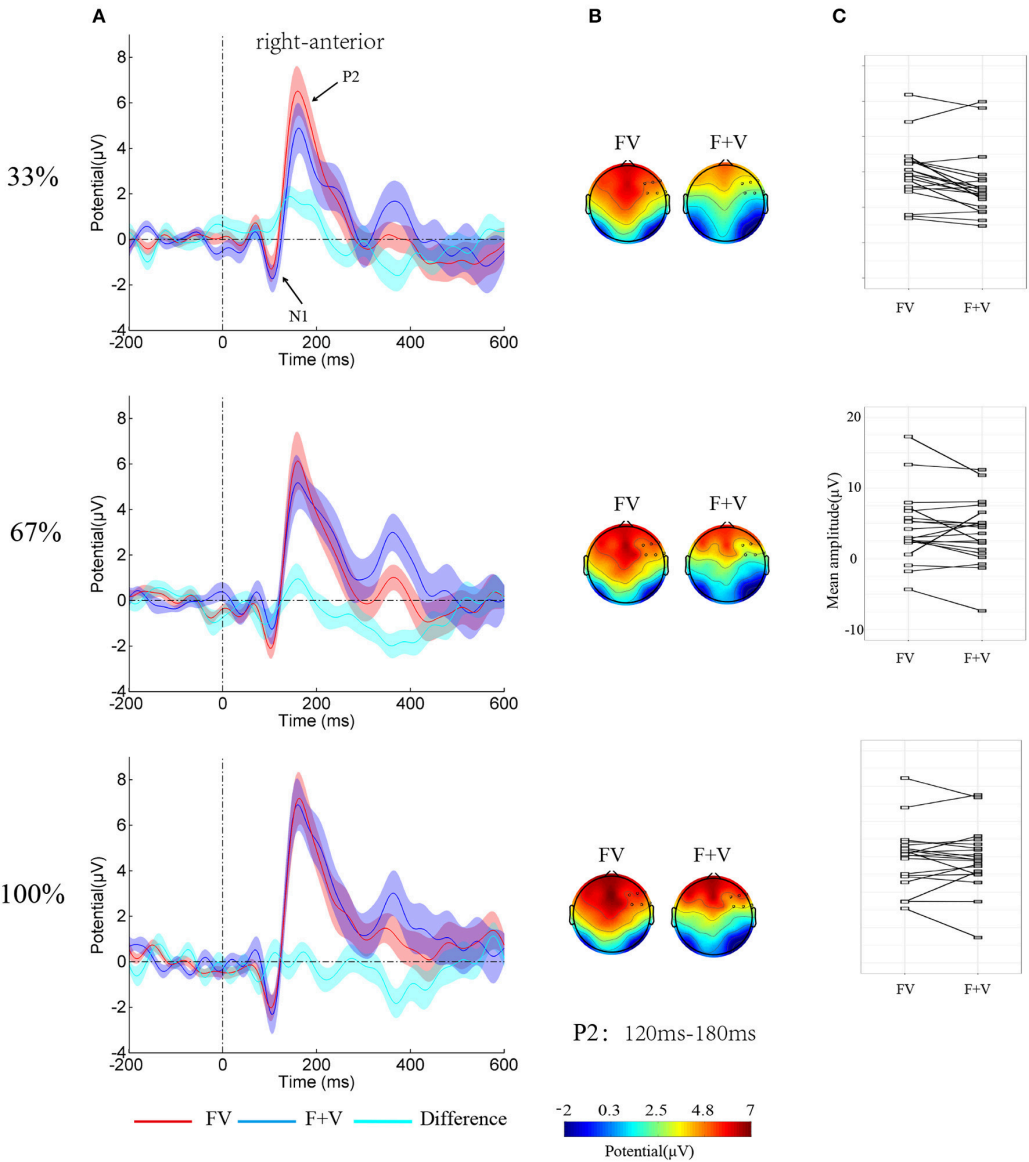
**(A)** Group-averaged ERPs over right-anterior (F4, F6, F8, FC4, FC6, and FT8) as function of Modality-type and Intensity. Shaded regions around waveforms represent standard error. **(B)** Topographies of bimodal and summed unimodal (facial + vocal) effect for P2 components. **(C)** The stripcharts with the value of the P2 amplitude over right-anterior to FV and F+V for each participant.

EEG data were preprocessed using EEGLAB (Delorme and Makeig, 2004), an open source toolbox running under the MATLAB environment. The data were down sampled at 250 Hz and then were high pass filtered at 0.1 Hz and low pass filtered at 40 Hz. After re-referenced offline to bilateral mastoid electrodes, the data were segmented to 1,000 ms epochs time-locked to the stimuli onset, starting 200 ms prior to stimuli onset. The epochs data were baseline corrected using the 200 ms before the stimuli onset. EEG epochs with large artifacts (exceeding ±100 μV) were removed and channels with poor signal quality were interpolated. Using the independent component analysis algorithm (Makeig et al., 2004), trials contaminated by eye blinks and other artifacts were detected and automatically excluded with the toolbox ADJUST (Mognon et al., 2011). More than 85% of the trials (34.64 ± 1.37, 34.28 ± 1.33, 33.88 ± 1.61, 34.28 ± 1.48, 34.48 ± 1.53, 33.60 ± 1.36, 34.24 ± 1.36, 34.84 ± 1.37 and 33.80 ± 1.52 for nine levels, respectively) were retained in further analysis. ERP waveforms were computed separately as function of condition. The extracted average waveforms for each participant and condition were used to calculate grand-average waveforms (see Figure 3 for ERP).

Given the simultaneously presentation of facial and vocal expressions in the current study and the strong visual processing component in the perception of audiovisual stimuli (Paulmann et al., 2009; Jessen and Kotz, 2011), the visual N1 and P2 were selected as the component of interested. Following the previous study addressing visual components associated with multimodal emotion integration (Paulmann et al., 2009) and visual inspection of the grand ERP waves, 80–120 ms and 120–180 ms were defined for N1 and P2, respectively. Since the N1 and P2 is found at anterior electrode-sites (Federmeier and Kutas, 2002; Paulmann et al., 2009), the anterior electrode-sites, were clustered as region of interest (Left anterior: F3, F5, F7, FC3, FC5, and FT7; middle anterior: F1, FZ, F2, FC1, FCZ and FC2; right anterior: F4, F6, F8, FC4, FC6 and FT8; middle central: C1, CZ, C2, CP1, CPZ, and CP2). To exam the influence of intensity on bimodal emotion integration, bimodal effect (FV), and summed unimodal effect (F + V) were calculated and tested with the additive model (Besle et al., 2004; Stevenson et al., 2014) by carrying out repeated measures ANOVA with *Modality-type* (FV vs. F+V), *Intensity*, and *SROI* as within-subject factors.

## Result

### Behavioral performance

The descriptive results of the rates of response anger and reaction times were depicted in Figure 2A. The two-way repeated measures ANOVA on rates of response anger showed significant main effect *Intensity* [*F*_(2, 38)_ = 74.97, *p* < 0.001, η^2^_*p*_ = 0.80], with the rates of response anger higher for 100% (0.90 ± 0.03) than for 67% (0.83 ± 0.04, *p* < 0.01) and 33% (0.48 ± 0.06, *p* < 0.001), and higher for 67% than for 33% (*p* < 0.001). Also significant was the main effect of *Modality* [*F*_(2, 38)_ = 8.71, *p* < 0.01, η^2^_*p*_ = 0.31], with the rates of response anger was higher for bimodal (0.81 ± 0.04) than for facial (0.72 ± 0.05, *p* < 0.01) and vocal (0.69 ± 0.05, *p* < 0.001), while the latter two conditions did not show significant differences (*p* > 0.1). However, as can be seen in Figure 2B, the integration analysis on rates of response anger showed no significant facilitation effects were observed under intensity levels.

Similarly, a two-way repeated measure ANOVA on reaction times revealed a significant main effect of *Modality* [*F*_(2, 38)_ = 51.24, *p* < 0.001, η^2^_*p*_ = 0.73], a significant main effect of *Intensity* [*F*_(2, 38)_ = 25.72, *p* < 0.001, η^2^_*p*_ = 0.58], and two-way interaction of *Modality* × *Intensity* [*F*_(4, 76)_ = 7.29, *p* < 0.01, η^2^_*p*_ = 0.28]. Simple effect analysis show that the reaction times were faster for 100% (634 ± 45 ms) than for 67% (719 ± 52 ms, *p* < 0.01) and 33% (769 ± 60 ms, *p* < 0.001) under vocal condition, while the latter two were not significantly differentiated (*p* > 0.1). In facial condition, the reaction times were faster for 100% (531 ± 33 ms) than for 67% (546 ± 37 ms, *p* < 0.01) and 33% (622 ± 50 ms, *p* < 0.01) under vocal condition, while the former two were not significantly differentiated (*p* > 0.1). The reaction times were faster for 100% (512 ± 39 ms) than for 67% (543 ± 40 ms, *p* < 0.01) and 33% (654 ± 52 ms, *p* < 0.001) under bimodal condition, and faster for 67% than for 33% (*p* < 0.001). However, as can be seen in Figure 2C, the integration analysis on reaction times showed no violation of the race model prediction over all quantiles of the reaction time distribution across three intensity levels.

### ERP results

The analysis on N1 amplitudes showed a significant two-way interaction of *Modality-type* × *SROI* [*F*_(3, 57)_ = 3.64, *p* < 0.05, η^2^_*p*_ = 0.16]. Simple effect analysis showed no significant integration effect of *Modality-type* for each *ROI* (*ps* > 0.05). However, The analysis on the P2 amplitudes showed a significant main effect of *Modality-type* [*F*_(1, 19)_ = 10.12, *p* < 0.01, η^2^_*p*_ = 0.35], a significant main effect of *Intensity* [*F*_(2, 38)_ = 7.02, *p* < 0.01, η^2^_*p*_ = 0.27], a significant two-way interaction of *Modality-type* × *SROI* [*F*_(3, 57)_ = 14.79, *p* < 0.01, η^2^_*p*_ = 0.44], and a significant three-way interaction of *Modality-type* × *Intensity* × *SROI* [*F*_(6, 114)_ = 3.11, *p* < 0.05, η^2^_*p*_ = 0.14]. To break down the three-way interaction, separate ANOVAs were performed for each *ROI*. A significant interaction between *Intensity* and *Modality-type* [*F*_(2, 38)_ = 3.61, *p* < 0.05, η^2^_*p*_ = 0.16] was found over *right-anterior area*, while no significant interactions were observed over other areas. Simple effect analysis showed that the amplitude larger for FV (4.77 ± 0.93 μV) than for F+V (3.07 ± 1.00 μV, *p* < 0.01). However, the ANOVAs for 67% and 100% did not reveal any significant effects of *Modality-type*.

## Discussion

The present study tested the modulation of emotional intensity on facial-vocal emotion, thereby examined whether bimodal emotion integration follows the principle of IE. It was found that the rates of response anger and reaction speed increased as emotional intensity across modalities. Critically, the P2 amplitudes elicited by bimodal condition were larger than the sum of two unimodal conditions for low emotional intensity stimuli, but nor for middle or high intensity stimuli. The significance of these findings will be addressed in the following discussion.

The rates of response anger and reaction speed for anger discrimination increased as the augment of emotional intensity across modalities. These findings were consistent with emotional intensity effect in facial emotion decoding, in which emotional intensity significantly correlated with classification of emotional expression (Herba et al., 2007; Dunning et al., 2010). The current findings were also in line with the study in vocal emotion (Juslin and Laukka, 2001; Wang et al., 2015), which reported that portrayals of the same emotion with different levels of intensity were decoded with different degrees of accuracy. Extending these studies, the current study reported the emotional intensity effect in bimodal emotion decoding.

One main findings of the current study is that the P2 amplitudes elicited by bimodal condition were larger than the sum of two unimodal conditions. P2 component has been repeatedly reported to be the neural marker of bimodal emotional integration during time range. For instance, Jessen and Kotz (2011) reported a larger P2 for bimodal condition than for vocal condition. Balconi and Carrera (2011) found that congruent audiovisual emotional stimuli elicited larger P2 compared to incongruent ones. However, several other studies reported a reduced P2 for congruent audiovisual emotional stimuli than for incongruent ones (Pourtois et al., 2002; Kokinous et al., 2015) and a suppression P2 components in the bimodal compared with the unimodal emotions (Pourtois et al., 2002; Paulmann et al., 2009; Kokinous et al., 2015). While the former line of studies interpret the larger P2 amplitude for congruent bimodal emotional expressions as a symbol of augment of emotional salience, the latter line of studies attribute the suppression P2 components in the bimodal vs. unimodal expressions to redundancy of multimodal stimuli. Obviously, the present findings are in line with the first line of studies (Jessen and Kotz, 2011; Schweinberger et al., 2011). Based on the fact that the visual P200 has been functionally linked to the emotional salience of a stimulus (Schutter et al., 2004; Paulmann et al., 2009), the present result suggested that the increase of P2 amplitudes for bimodal stimuli results from that fact that bimodal emotional cues increase the emotional salience embedded in the stimuli. Moreover, the current findings allow us speculate that the current differentiation in P2 components suggests the bimodal emotional integration take place during the stage of emotional information derivation.

More critically, the integration effect only existed in low emotional intensity stimuli, but not for high intensity stimuli, suggesting a modulation of emotional intensity on the facial-vocal emotion integration. Consistent with the previous studies (Leppänen et al., 2007; Wang et al., 2015), the behavioral and brain responses linearly varied as a function of intensity levels regardless of sensory modalities. However, when test the integration effect as a function of emotional intensity, only the bimodal integration for stimuli with low emotional intensity reached the statistical significant level, suggesting the integration of bimodal emotional cues follows the IE principle. This finding is consistent with the previous findings that multimodal emotions elicited earlier N1 than unimodal emotions under high noisy background but not low noisy background (Jessen et al., 2012) and bimodal cues affect each other when the target modality was less reliable (Collignon et al., 2008). However, the present study extended the previous studies by showing that emotional intensity modulates bimodal emotional integration.

Inconsistent with the previous studies (Jessen et al., 2012; Kokinous et al., 2015, 2017), we did not find significant integration effect on behavioral responses. Moreover, although the N1 amplitude appeared to be smaller for the bimodal condition than for the sum of the unimodal condition, it failed to reach statistically significant level. This result is inconsistent with previous finding that the N1 amplitude for the bimodal condition is smaller than for vocal condition (Jessen and Kotz, 2011; Jessen et al., 2012; Kokinous et al., 2015). However, this result is consistent with the study by Paulmann et al. (2009) and Balconi and Carrera (2011), which only reported significant integration effect during P2 time range. One possible explanation for this discrepancy might be that the current study tested the integration effect with superadditivity, which is stricter than comparing brain response for bimodal condition with unimodal condition (Jessen and Kotz, 2011; Jessen et al., 2012; Kokinous et al., 2015). Another possibility is that we presented facial expression with full emotionality simultaneously with the vocal emotions. As the previous studies reporting integration effect in N1 was locked to the onset of the auditory stimuli, whether there is a discrepancy on the visual and auditory worth further exploration.

## Conclusion

In sum, the present study manipulated the emotional salience of the stimulus itself and tested the facial-vocal emotional integration with the criterion of superadditivity. The rates of response anger and reaction speed increased as emotional intensity across modalities. More importantly, the P2 amplitudes were larger for bimodal expressions than for the sum of facial and vocal expressions for low emotional intensity stimuli, but not for middle and high emotional intensity stimuli. These findings indicated that bimodal emotional integration appears to be affected by emotional intensity and integration of facial-vocal angry expressions follows the principle of IE.

## Ethics statement

This study was carried out in accordance with the recommendations of Key Laboratory of Behavior and Cognitive Psychology in Shaanxi Province, Shaanxi Normal University with written informed consent from all subjects. All subjects gave written informed consent in accordance with the Declaration of Helsinki. The protocol was approved by the Shaanxi Normal University.

## Author contributions

Conceived and designed the experiments: ZP and XC. Performed the experiments: ZP and XL. Contributed materials/analysis tools: ZP and XL. YL helped perform the analysis with constructive discussions. ZP and XC performed the data analyses and wrote the manuscript.

### Conflict of interest statement

The authors declare that the research was conducted in the absence of any commercial or financial relationships that could be construed as a potential conflict of interest.

## Footnotes

^1^Where *p*(V) is the rates of response anger for vocal expressions and *p*(F) is the rates of response anger for facial expressions.
