# Supplementary material for: Emotional Intensity Modulates the Integration of Bimodal Angry Expressions: ERP Evidence
Source: Front Neurosci. 2017 Jun 21;11:349. doi: 10.3389/fnins.2017.00349 (PMC5478688; doi:10.3389/fnins.2017.00349)
Supplement: Supplementary file 1 [file Presentation1.pdf]

## Material development

Facial and vocal expressions were produced by two trained actors (one male) of Mandarin Chinese, who were paid for their participation at the rate of ￥200/h. The actors were instructed to express anger, sad, happiness and neutral through both facial and vocal expressions. They were asked to sit as still as possible to keep head movement to minimum. Seven interjections “嗯/en/，啊/ah/，嘿/hei/，噢/o/，哇/wa/，喂/wei/，哟/yo/” were recorded as they contain no linguistic information. The actors sat in front of the camera, at a distance of 100 cm away from camera in a recording chamber, ensuring that the camera (Canon EOS 600D) captured a directly frontal view of the face. Sound information was transmitted via a microphone.

Video output was split into different clips. The maximum mouth opening pictures were chosen for facial expressions. To minimize contrast differences, the hair were removed and all facial expressions were presented in grayscale. The audio recording for each clip was edited in Adobe Premiere (Adobe Systems Incorporated, San Jose, CA) to reduce noise. The vocal expressions were acoustically analyzed using Praat (Boersma & Weenink, 2006) and the analysis showed that each category of vocalizations have classical acoustic features (see Table 1 for details) reported in the previous study (Banse & Scherer, 1996). Then, the facial and vocal expressions were presented simultaneously to get bimodal emotional stimuli.

Table 1 The acoustic features of the vocalizations (M ±SD) as a function of emotional category.

| | F0(Hz) | Intensity(dB) | Duration(ms) |
|---|---|---|---|
| sad | 265 ±58 | 74 ±1.6 | 589 ±87 |
| anger | 381 ±51 | 76 ±1.8 | 407 ±75 |
| happy | 358 ±54 | 75 ±3.5 | 618 ±167 |
| neutral | 235 ±23 | 74 ±1.4 | 327 ±38 |

Moreover, twenty eight participants were asked to classify the emotionality conveyed by the stimuli through forced choice among four emotion categories (anger, happy, sad and neutral). The results found that each category was classified with more than 60% right (see Table 2 for details), significantly higher than change level (25%), indicating that the recorded materials expressed the target emotions.

# Table 2 The accuracy (M ±SD) as a function of emotional categories (anger, happy, sad, and neutral) and modality (facial, vocal, and bimodal).

| | facial | vocal | bimodal |
|---|---|---|---|
| sad | 0.66 ±0.17 | 0.81 ±0.14 | 0.95 ±0.06 |
| anger | 0.86 ±0.09 | 0.80 ±0.10 | 0.96 ±0.06 |
| happy | 0.98 ±0.03 | 0.69 ±0.16 | 0.98 ±0.04 |
| neutral | 0.71 ±0.15 | 0.63 ±0.20 | 0.80 ±0.11 |

**References**

Banse, R., & Scherer, K. R. (1996). Acoustic profiles in vocal emotion expression. *Journal of personality and social psychology*, 70(3), 614.

Boersma, P., & Weenink, D. (2006). *Praat: doing phonetics by computer* (Version 4.4. 11)[Computer program]. Retrieved February 23, 2006.
